# Books Reviewed

**Published:** 1867-01

**Authors:** 


### Books Reviewed.

Medical Jurisprudence. By ALFRED SWAIN TAYLOR, with Notes and References to American Decisions, by Clement Penrose. Philadelphia: Henry C. Lea, 1866.

The appearance of a new American edition of this work is not an occasion to speak at all of its merits or defects, since the intelligent portion of the profession—all that portion, at least, who read the Buffalo Medical and Surgical Journal—are sufficiently familiar with the established facts of its standard volume. It is, however, an occasion to speak of this, and similar works, and to urge the profession to a higher appreciation of their practical importance. Whoever carefully notices medical testimony as it is given, not unfrequently in the courts of justice, will see the necessity of increased attention in the profession to legal medicine. All the different branches of medical science, anatomy, physiology, chemistry, surgery, botany and physics lend their aid to the requirements and proper administration of law, and a familiarity with their correct and truthful application, increases the usefulness of the physician, and at the same time reflects upon them deserved credit.

This edition of Taylor's Medical Jurisprudence has been improved in many respects; new additions have been made in the articles on noxious animal food, trichiniasis, sexual malformation, insanity as affecting civil responsibility, suicidal mania and suicide, and life insurance. Into the present edition Dr. Penrose has also introduced numerous references to American practice and decisions, thus greatly increasing the value of the work, at least, to the profession of this country. Some physicians are inclined to look upon medico-legal practice as an unimportant and unnecessary addition to their professional duties; but there are few but will often find themselves in situations from the accidental occurrence of cases, where it is utterly impossible to shift the responsibility. Neglect of reasonable qualification is liable to bring public censure, will certainly meet with exposure of deficiencies, and often lead to punishment of the innocent or acquittal of the guilty.

This woık, as now presented to the profession, appears to lack nothing in completeness; is full and wide in its range of defects, and worthy the high rank it holds as a standard work.

---

Provision for the Insane Poor of the State of New York. BY CHARLES A. LEE, M. D.

This is a very able paper upon the subject of proper care of the insane and the advantages of the "Asylum and Cottage plan" over other and moro expensive arrangements, such as are now generally adopted in the United States. This plan consists in opening a home, and placing it under the care of a "married couple of well-tried, faithful, skillful attendants, with a family circle of convalescents of both sexes." A great many other plans are described and their merits discussed. Prof. Lee has given great attention to the subject, and for years both at home and in foreign countries has been observing and comparing the advantages of all systems, so that his present opinions are entitled to, and will command the highest respect.

Report to the War Department upon the Prevailing Diseases of the United States, by J. H. Baxter, M. D., Surgeon and Brevet Colonel U. S. Volunteers, and Chief Medical Officer Provost Marshal General's Bureau.

This report is worthy of special attention; it is based upon data probably more comprehensive and full than any other Government has as yet furnished. We have exhibited in tabular form a complete view of the physical condition of the nation; and when these accumulated facts shall have been fully digested, they will, it is thought, throw much light upon the causes of many of the more common diseases of mankind. These tables are 158 in number, and show facts by very extensive statistical proof; all the facts which could be gained from examination of the enlisted and drafted men, during the late war, have been carefully and appropriately embodied in this form, and thus made available for future use. We have no space to even indicate in the briefest manner the nature and extent of this report. It constitutes a large volume, and furnishes an amount of statistical information beyond all estimate in value and importance. It is at present published only in the Public Documents of the War Department, but it is to be hoped that whatever portions of it can be made available for medical knowledge, will be furnished the profession in more available form. Dr. Baxter, with the true instincts of an educated physician, has not forgotten to contribute what came within his reach for the increase of medical knowledge, and what to others would have appeared only as the dry and uninteresting statistics of the recruiting business, under his masterly supervision, show upon a grand scale the most important facts connected with the physical history of mankind. The War Department, the medical profession, and the public, are under the greatest obligations to him since the labor of this report is immense, and its value is in proportion to the work of preparing it.

---

Transactions of the Medical Society of the State of Pennsylvania, at its Seventeenth Annual Session.

This volume comprises the report of the proceedings; reports of county medical societies; report of standing committees; catalogue; constitution, by-laws, etc.,etc. The County Society reports are valuable as a history of disease in the State, and many of them are very well written and instructive papers. One communication was received, being a report made to the Alleghany County Medical Society by Dr. A. J. Davis, of East Liberty, in regard to an abnormal pregnancy of *forty-nine* years duration. This case is remarkable, and will appear in full in the present or next number of our journal.

---

On Excision of the Superior Maxilla—Report of a case, with remarks on certain tumors of this bone. By Wm. R. Whitehead, M. D., (Univ. of Paris) formerly of the New York Medical College.

A well written report of a judiciously planned, skillfully executed operation for removal of tumor on the superior maxilla. The tumor before its removal and the girl after its removal are represented, showing most remarkable results; the features are perfect, and not the least appearance of deformity is visible. Remarks upon tumors in this vicinity are instructive, and the whole report reflects much credit upon the author.

Aitkins' Science and Practice of Medicine.

We have received the first volume of this work, which, when completed, will comprise the most comprehensive range of topics of any recent treatise upon the practice of medicine. Volume two is nearly ready, and will contain a copious index to both volumes.

The title to this work indicates somewhat its character; the science of medicine, evidently proposes a fuller consideration of the pathology of disease and the philosophy of cure than is usually attempted in our text-books on the practice of medicine. Within the last few months the department of practical medicine has been advanced by the publication of stardard works which may safely be followed in the treatment of disease. Until within this short period it could not be said that any such standard work existed; we have heretofore believed that, whoever followed faithfully the teachings even of the best authors in practical medicine, would do a great deal more harm than good. The introduction to the profession of these works will constitute a new era in medical practice.

This comprehensive work by Aitkins has excellencies which at present we have not space to suitably notice, and when the 2d volume reaches us, another opportunity will be presented to speak of its merits more in detail. It may be sufficient for the present to say, that this work will constitute in itself a library of practical medicine, and should find a place in the office of every practicing physician.

---

Jones on Nervous Disorders.

This volume contains a special notice of the nervous disorders usually mentioned by authors, with full description of the causes, symptoms, differential diagnosis, and treatment. The author writes in original style, and has views and opinions, which we have no doubt are peculiarly his own. He has had great opportunity to observe and treat these various affections at St. Mary's Hospital, and his opinions are worthy of great respect. He appears to entertain a profound belief in the almost supreme efficacy of drugs, and his illustrative cases show that the author believes in the controlling influence of medicine. In some respects the work seems of former date, with the older notions of disease and medicine, while in others it is fully up to the most recent pathological and therapeutical views. This book is valuable as a practical guide, and consists largely of illustrative cases, the study of which are exceedingly instructive.

---

Clinical Lectures by Prof. A. Von Græfe, on Amblyopia and Amaurosis and Extraction of Cataract. Translated from the German by Hasket Derby, M. D., Surgeon to the Massachusetts Charitable Eye and Ear Infirmary, etc., etc.

The translator has conferred a real favor upon the profession by introducing the author to the American medical public. A. Von Græfe is unexcelled in his department, and has enriched the science of ophthalmology by as much valuable discovery as any man in this department. The American profession will take deep interest in careful perusal of this translation. Its suggestions would be copied in full if space could admit of such notice. The translation was made for the Boston Medical and Surgical Journal, and the pamphlet is published from the same office, David Clapp & Son, 334 Washington street, Boston, Mass.

Bowman's Practical Chemistry. Philadelphia: Henry C. Lea, 1866.

The application of practical chemistry to practical medicine is becoming every year more and more apparent, and a text-book adapted to the wants of the physician and student upon practical chemistry will be received with favor. This is a text-book of practical chemistry, with over one hundred illustrations, and eminently suited to the purposes for which it is designed. We have not space to speak of its merits in detail, but with what opportunity has been afforded for examination we believe it possesses unusual attractions, which will be better appreciated by those who are allowed to make a more thorough examination. It contains a great variety of chemical facts and experiments; is beautifully illustrated; has copious index, and full glossary of medical terms. It could not be better, or contain more in its present size.

---

Transactions of the Vermont Medical Society.

This volume contains proceedings of the Society; address of the President, O. F. Fassit, M. D.; statistics of diptheria in Vermont, by L. Richmond, M. D.; criminal abortion, by Wm. McCollum, M. D.; cerebro spinal meningitis, by B. F. Ketchum, M. D.; morbus coxarius, by Benjamin Fairchild, M. D.; statistical tables, by C. P. Frost, M. D., and some other reports upon local matters. We congratulate the profession in Vermont upon the appearance and value of this volume, which is highly creditable to it. The treatment of morbus coxarius by Dr. Fairchilds is so remarkable that we shall hereafter give it a fuller notice.

---

Notes on Epidemics. for the Public, by Francis Edmund Anstie, M. D., F. R. C. P. Philadelphia: J. B. Lippencott & Co., 1866.

This is a scientific, beautifully published, little volume, designed for the public; and could not have been better adapted to its object. While it is within the comprehension of the intelligent, and will instruct them in everything of which it treats, it is also suitable to the profession, and much of interest and importance will be found within its few pages. The causes of disease, its prevention and cure, are discussed with eminent ability, and the little volume contains more sense and sound reasoning than many larger works.

---

A Treatise on Vesico-Vaginal Fistula. By M. Schuppert, M. D., Surgeon of the Orthopædie Institute, New Orleans, La.

This is a pamphlet received some time since, which has received no full notice for want of space to do it justice. It is a monograph upon this subject, containing Definition, Causes, After Treatment, Cases, History of the Operation. The operation is beautifully illustrated in its various stages, and many useful suggestions made. The paper is worthy attentive perusal and nothing short of this can satisfy any one having interest in this operation.

---

Physician's Pocket Record.

We have received from the office of the Philadelphia Medical and Surgical Reporter, the most admirably planned, beautifully bound Pocket Record which has yet made its appearance. The table of contents was published in our last journal, so that all who desire can see at a glance how much may be comprised in a pocket record. The price is $1.50, which appears to us as vastly below the publishing cost, but we presume deficiencies, if any, will be made up by increased subscription to the Reporter, a weekly journal of great merit. Those wishing to obtain copies may address, Medical and Surgical Reporter, Philadelphia. Pa.

